# Expectations and Disappointments: The War and the Strikes

**Published:** 1919-02-22

**Authors:** 


### The Hospital

The Workers' Newspaper of Administrative Medicine and Institutional Life, Administration, National Insurance and Health.

No. 1707, Vol. LXV. SATURDAY, FEBRUARY 22, 1919. PRICE TWOPENCE

### EXPECTATIONS AND DISAPPOINTMENTS: THE WAR AND THE STRIKES.

During the long and trying strain of the war there naturally arose in all the belligerent countries not only a desire for peace, but also a pleasing anticipation that peace would bring with it both relief from anxiety and personal and national happiness. The expectation included benefits both positive and negative. In such an attitude of mind limitations and hindrances found little space. Peace was to set its seal on a restful and contented world, and enmities and struggles were to be stilled. That experienced and far-seeing minds discounted this picture is quite true, but while hostilities were still in progress and the issue doubtful it would not have been politic to emphasise this qualification, and, without question, the community generally looked forward with eager and confident hopefulness. The comradeship in arms was to be followed by a comradeship in industry, and the nation, disciplined by suffering and by a common effort in the cause of victory and right, was to take up with hearty goodwill and mutual helpfulness the rebuilding of its corporate life. The cynic may have smiled at the simplicity of these sentiments, but it will hardly be doubted that they were one of the factors which contributed to union both in effort and in endurance. A sense of peril had some part in holding men together, but there was, in addition, a belief that from the unparalleled wrong and suffering would issue a brighter and a happier world.

The reality, it must be admitted, has in not a few respects disappointed these high hopes. The multitude of questions raised by the war on the plane of international politics and the complexities of many of these issues necessarily compel prolonged discussions, and definitely and satisfactorily to make peace is found to be a process painfully tedious as compared with the framing of our answer to the direct challenge of war. Conferences and commissions and committees, with their qualified and provisional findings, become somewhat irksome to spectators anxious for a prompt settlement and for an immediate enjoyment of the interpretation widely attached to the blessings of peace. Not only does the process seem long. It is shadowed also by a sense of uncertainty. Will these complex and half-concealed debates result in agreement? And, if so, what will be the character of this agreement, and in what measure and direction will it affect the nation and the individual? The air is filled with questions of this order, and daily they perplex and perturb men's minds. In war the line of duty was plainly marked. But in the present transition time there is no such clear line; or, if the line is distinct, it is less easy to follow. To suffer and be strong is a call full of stimulus. But to be patient and hopeful amidst uncertainties is much less obviously heroic, and the divided mind is apt to mean wavering conduct. Yet it is by steadiness in resolve and action that the nation has achieved victory. And it is by steadiness in resolve and action that it will achieve peace.

In some respects the situation at home appears to not a few even more full of disappointment than does the entangled and slow-moving effort towards international peace. To the stately and reluctant programme of diplomacy we have been accustomed by many experiences. But in domestic affairs we expect to find a greater haste, or, at least, a greater show of haste. Such an expectation is naturally the more acute after the long necessity for delay compelled by the war, and it is apt to have impatient modes of expression in those who have been held under severe strain. The reaction from tense nerves is a physiological process, and must be allowed for, even when it passes beyond safe and wise limits. Patience, in other words, must not fail, though confronted with the words or works of the impatient. The lesson just now has many an opportunity for application. Instead of the calm and happiness and content with which the anticipated peace was charged, the country seems distracted by dissatisfaction and ill-temper. Trade disputes leading to sudden and inconsiderate strikes darken both the hour and the prospect, and readily provoke angry and resentful judgments. There seems no sure leadership or commanding will, and the pessimists find ready to hand many demonstrations appropriate to their gloomy gospel. Nor are they content with the present position. On the contrary, they assure us that the existing disturbances are but the prelude to a great convulsion in which the social and economic order of the country will be broken in pieces. To judge from some of these voices, there are those who have learned nothing from the war, or, at the best, they have found in this experience a confirmation of their faith in the value and efficacy of force.

It is not likely that in these columns we have any apology to offer for violence or for defiance of the law of the land. Nor do we make excuses for actions which show an intention to penalise the community for the benefit of individual classes. Yet we are quite unable to join in the sweeping verdicts which are often presented to us. Crime and violence must be met by sternness, but some measure of patience is necessary before we indict large sections of our fellow-citizens, the great majority of whom have been loyal to the country in the time of its greatest stress and have submitted to sacrifices of no mean order. Doubtless extravagant expectations have grown up in certain directions, and, as realisation of even moderate hopes is slow, there is impatience and disappointment. But there is in this country a sound basis of common-sense and moderation, and this, we are convinced, will keep the nation steady. Let the better instructed set a good example, and not by hasty and half-formed judgments illustrate the impatience which they condemn in others.

